# Phasing out India ink: the case for the CrAg LFA in modern practice

**DOI:** 10.1128/spectrum.01531-25

**Published:** 2025-12-05

**Authors:** Sejal Morjaria, Derick Iturralde, Brenden Clarck, N. Esther Babady

**Affiliations:** 1 Infectious Disease Service, Department of Medicine, Memorial Sloan Kettering Cancer Center 5803, New York, New York, USA; 2 Clinical Microbiology Service, Department of Pathology and Laboratory Medicine, Memorial Sloan Kettering Cancer Center 5803, New York, New York, USA; The George Washington University School of Medicine and Health Sciences, Washington, DC, USA

**Keywords:** diagnostic stewardship, microbiology

## Abstract

Cryptococcal meningitis remains a life-threatening infection, particularly in immunocompromised patients, and timely, accurate diagnosis is essential. In a retrospective review of 489 cerebrospinal fluid samples at a tertiary cancer center, the India ink stain demonstrated low sensitivity (42.9%) compared to lateral flow antigen (100%) and multiplex PCR (100%) methods. These findings support replacing the outdated, labor-intensive India ink test with more accurate, rapid diagnostics to improve detection and patient outcomes.

**IMPORTANCE:**

The India ink test has historically been a cornerstone of cryptococcal diagnosis, but its low sensitivity and reliance on subjective interpretation make it increasingly obsolete in modern practice. Continued reliance on India ink risks delayed or missed diagnoses, particularly in immunocompromised patients where early detection is critical. In contrast, the cryptococcal antigen lateral flow assay (CrAg LFA) and other modern diagnostics provide superior sensitivity, ease of use, and reproducibility. Phasing out India ink in favor of the CrAg LFA is essential to align clinical practice with contemporary diagnostic standards and to improve patient outcomes.

## OBSERVATION

Cryptococcal meningitis is a serious fungal infection of the central nervous system caused by *Cryptococcus neoformans*. It carries a high mortality risk, especially in immunocompromised individuals (both HIV and cancer patients) if not promptly diagnosed and treated (1, 2). The use of India ink for the diagnosis of *Cryptococcus* spp. has long been a standard diagnostic tool in microbiology laboratories (3, 4). However, this method is increasingly being replaced due to advances in diagnostic technology, such as the cryptococcal antigen lateral flow assay (CrAg LFA), and molecular multiplex meningitis/encephalitis (M/E) panels (BioFire). The India ink stain highlights the capsule of *Cryptococcus* and requires a trained medical laboratory scientist to prepare and interpret the sample, with accuracy heavily dependent on the examiner’s skill and subject to interobserver variability (5). Additionally, the India Ink test typically requires a high fungal burden (>1,000 CFU/mL) for detection, increasing the risk of errors such as false negatives (6, 7). Consequently, the India ink test has relatively low sensitivity, ranging from 50% to 80%, and may fail to detect *Cryptococcus* infection, especially in cases when the fungal burden is low (8–10). Despite these limitations, India Ink remains in routine use at many institutions, including our own at Memorial Sloan Kettering Cancer Center, and it continues to be featured in proficiency testing challenges by the College of American Pathologists (11).

To better understand the current role of this assay, we evaluated its clinical performance in our laboratory. We evaluated the clinical utility of India ink staining by reviewing its performance in our laboratory between January 2018 and August 2024. During this period, 457 cerebrospinal fluid (CSF) samples from 439 patients were tested for cryptococcal infection using India ink stain, CrAg LFA, fungal culture, and M/E multiplex PCR panel. The CrAg LFA was performed using the IMMY CrAg LFA kit (Immuno-Mycologics, Norman, OK) according to the manufacturer’s instructions. Briefly, CSF specimens were mixed with specimen diluent, and a lateral flow strip was inserted; results were read visually at 10 minutes. Semiquantitative titers were obtained by serial twofold dilutions (up to 1:2,560) as per the package insert (12, 13).

**Fig 1:**
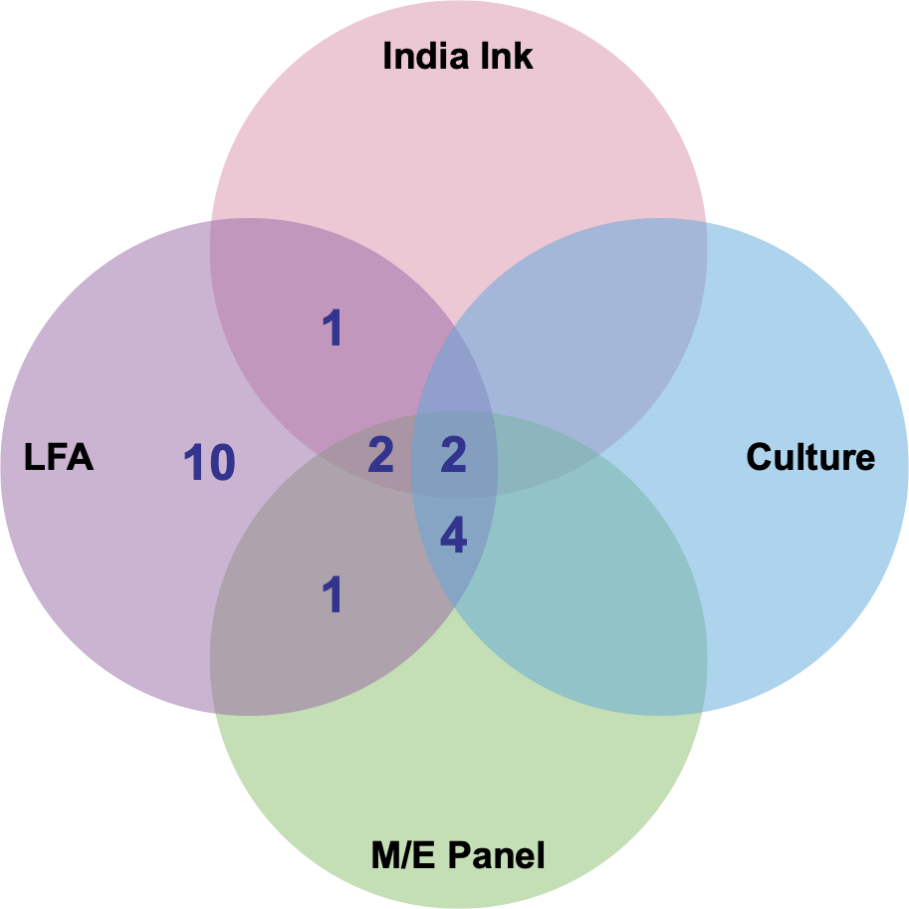
Venn diagram of diagnostic tests positive for *Cryptococcus neoformans*. A total of 457 samples were tested by all four methods, with 20 samples resulting as positive, including 20 samples positive by the cryptococcal antigen lateral flow assay (LFA), 9 positives by the meningitis/encephalitis (M/E) panel, 6 positives by culture, and 5 positives by India ink.

Among the 457 CSF samples reviewed, all positive results were identified by the CrAg LFA (*n* = 20); of these, 9 were also positive by the M/E panel, 6 by fungal culture, and 5 by India ink stain (Fig. 1). CSF specimens in this study were obtained both at the time of initial diagnosis and, in some cases, during treatment follow-up to monitor response or evaluate ongoing symptoms. Among the nine patients who tested positive by LFA, four contributed a single CSF sample; two contributed two samples each; two contributed three samples each; and one contributed six samples, accounting for the difference between the number of unique patients and the total number of CSF specimens analyzed.

Of the 439 patients from whom CSF was collected, only one was HIV positive and receiving antiretroviral therapy; the remainder were HIV negative, all with underlying malignancies, most commonly hematologic. None had received antifungal therapy prior to CSF collection, ensuring that diagnostic test sensitivity was not confounded by prior treatment. All positive results were confirmed as true cases of cryptococcal infection based on expert chart review by an infectious disease physician (author, S.M.), who assessed clinical presentation, treatment, and response, often in conjunction with infectious disease consultation. Notably, all cases were due to *Cryptococcus neoformans*; no cases of *Cryptococcus gattii* were identified.

Using culture as the gold standard, the sensitivity and specificity of India Ink was 42.8% and 99.4%, respectively, compared to 100.0% and 94.7% for the LFA and 100.0% and 99.3% for the M/E panel (Table 1). Notably, the sensitivity of India ink in our cohort was lower than the commonly cited range above. Several factors likely contributed to this reduced sensitivity. Because specimens were centrifuged prior to slide preparation, lack of centrifugation did not account for the diminished yield. Rather, the low fungal burden in many of our patients, particularly those with cancer and without HIV, likely contributed to false-negative India ink results (14–17).

**TABLE 1:** Performance characteristics of India ink, lateral flow, and the M/E multiplexed molecular panel compared to fungal culture^*a*^

	Total number	Culture positive	Culture negative	Sensitivity (%) (95% Cl)	Specificity (%) (95% Cl)
India ink positive	6	3	3	42.8 (15.8%–75.0%)	99.4 (98.2%–99.8%)
India ink negative	483	4	479		
CrAg LFA positive	26	7	19	100 (64.5%–100.0%)	94.7 (93.9%–97.6%)
CrAg LFA negative	463	0	463		
M/E panel positive^*b*^	9	6	3	100 (61%–100.0%)	99.3 (98.1%–99.8%)
M/E panel negative	448	0	448		

^*a*^ Total number of meningitis/encephalitis (M/E) panels: 457.

^*b*^ An additional 16 M/E panels were positive for targets other than *Crypotococcus *and counted as negative.

The discrepancy between the cryptococcal antigen LFA and the M/E panel (20 LFA positive versus 9 M/E positive) likely reflects the superior sensitivity of the LFA. In subacute or treated cases, fungal burden may be low and fall below the detection threshold of the M/E panel, whereas the LFA can still reliably detect cryptococcal polysaccharide antigens. Although not the primary focus of this study, these findings underscore an important limitation of molecular testing in certain clinical contexts (1).

Beyond the discrepancy with the M/E panel, a comparable limitation was evident when evaluating culture and India ink against the LFA. In our study, 14 CSF specimens were culture negative but LFA positive, with clinical features consistent with cryptococcal meningitis, underscoring the limitations of culture in early or low-burden disease. Of these, India ink was negative in 11 cases and positive in 3, with the positives occurring only at high antigen titers (≥1:2,560) apart from a single lower-titer sample, highlighting the test’s poor sensitivity and its limited utility compared with antigen-based assays. These findings emphasize that the CrAg LFA is more sensitive than both culture and India ink, particularly when the fungal burden is low (18, 19).

While the CrAg LFA has largely replaced India ink due to its high sensitivity, simplicity, and availability, India ink may retain limited value in high-burden cases or when an LFA post-zone effect is suspected, and in institutions with limited on-site testing, it may serve as a rapid but low-yield adjunct while confirmatory testing is performed off-site (12, 15).

Overall, our results support the use of the CrAg LFA as the preferred frontline diagnostic test for cryptococcal meningitis while recognizing that India ink and culture retain limited utility in specific high-burden or confirmatory contexts.
